# Expression of Functional MHC Class II Molecules By a Mouse Pro-B Cell Clone

**DOI:** 10.1155/1995/10359

**Published:** 1995

**Authors:** Suzanne Lombard-Platet, Amanda G. Fisher, Valérie Meyer, Rhodri Ceredig

**Affiliations:** 1 Laboratoire de Génétique Moléculaire des Eucaryotes du CNRS et Unité 184 de I'INSERM Institut de Chimie Biologique 11 rue Humann Strasbourg 67085 France; 2 MRC Clinical Sciences Center Royal Postgraduate Medical School Hammersmith Hospital Ducane Road London W12 ONN United Kingdom

**Keywords:** MHC class II molecules, pro-B cells, antigen processing

## Abstract

We describe here the G12 pro-B cell clone that has been isolated from an IL-7 transgenic mouse. This clone has the phenotype B220^+^, BP-1^+^ , HSA ^+^, CD43^+^ λ5^+^ , and CD25^-^, and has its Ig locus in a germline configuration. G12 cells spontaneously express cell-surface MHC class II molecules, although to a much lesser extent than the mature M12.4.1 B-cell lymphoma. G12 cells can process and present the native Hen Egg Lysozyme (HEL) to an MHC class II-restricted T-cell hybridoma. The efficiency of presentation is inferior to that obtained with M12.4.1 cells. This is the first report where a pro-B cell can serve as APC in an MHC class II-restricted presentation.


*Developmental Immunology*, 1995, Vol 4, pp. 85–92
Reprints available directly from the publisher
Photocopying permitted by license only

© 1995 Harwood Academic Publishers GmbH
Printed in Singapore

### Expression of Functional MHC Class II Molecules by a Mouse Pro-B Cell Clone

SUZANNE LOMBARD-PLATET,*† AMANDA G. FISHER,‡ VALÉRIE MEYER,† and RHODRI CEREDIG†

†*Laboratoire de Génétique Moléculaire des Eucaryotes du CNRS et Unité 184 de l'INSERM, Institut de Chimie Biologique, 11 rue Humann, 67085 Strasbourg, France*
‡*MRC Clinical Sciences Center, Royal Postgraduate Medical School, Hammersmith Hospital, Ducane Road, London W12 ONN, England*

We describe here the G12 pro-B cell clone that has been isolated from an IL-7 transgenic mouse. This clone has the phenotype B220$^+$, BP-1$^+$, HSA$^+$, CD43$^+$, λ5$^+$, and CD25$^-$, and has its Ig locus in a germline configuration. G12 cells spontaneously express cell-surface MHC class II molecules, although to a much lesser extent than the mature M12.4.1 B-cell lymphoma. G12 cells can process and present the native Hen Egg Lysozyme (HEL) to an MHC class II–restricted T-cell hybridoma. The efficiency of presentation is inferior to that obtained with M12.4.1 cells. This is the first report where a pro-B cell can serve as APC in an MHC class II–restricted presentation.

KEYWORDS: MHC class II molecules, pro-B cells, antigen processing.

### INTRODUCTION

The differentiation of B-cell precursors in the bone marrow occurs through ordered events that comprise the successive loss or acquisition of various cell-surface markers as well as the progressive rearrangement of Ig genes. Several nomenclatures have been proposed (reviewed by Hardy et al., 1994 and Rolink et al., 1994). According to the classification of Rolink et al. (1994), the most immature B cells, called pro-B cells, sequentially mature into pre-BI, large pre-BII, small pre-BII, and immature B cells. Pro-B cells express low levels of the B220 (CD45) antigen, and are dependent upon a combination of IL-7 and cell contact with stromal cells for their *in vitro* growth. At this stage, the cells are CD43-positive, and the surrogate light chain (SL), comprised of the λ5 and Vpre-B molecules, is expressed and associated with a complex of cell-surface glycoproteins (Rolink et al., 1994). The transition between pro-B and pre-BI cells is marked by the rearrangement of $D_H$ to $J_H$ genes. At the large pre-BII cell stage, cells contain cytoplasmic μH chain, do not express cell-surface SL anymore, become CD25-positive, and are able to grow *in vitro* in the presence of IL-7 alone. The subsequent small pre-BII cells lose expression of the CD43 antigen. They then become immature B cells with surface expression of the μH chain associated with light chains and do not respond to IL-7 anymore. Mature B cells then enter the periphery and coexpress cell-surface IgM and IgD.

One major point of conflict concerning the B-cell differentiation pathway is the timing of MHC class II molecules expression. In the periphery, mature B cells express class II molecules until the plasma-cell stage, at which point the expression is down-regulated. Acquisition of class II molecules by developing mouse B cells in the bone marrow remains controversial. Previous studies using flow cytometry of bone marrow or fetal liver-derived *in vitro*-generated pre-B cells failed to demonstrate any MHC class II expression (Dasch and Jones, 1986; Rolink et al., 1991). Other studies, using Abelson murine leukemia virus-transformed or chemically transformed pre-B cell lines, showed that these cell lines failed to express class II molecules unless they were induced by IL-4 (Polla et al., 1986). However, recent reports have challenged this view. Significant amounts of class II molecules could be detected on bone-marrow-derived pre-B cells using a highly sensitive ELISA test (Miki et al., 1992) or multiparameter flow cytometry (Tarlinton, 1993; Hayakawa et al., 1994).

*Corresponding author.

In this report, we characterized a pro-B-cell clone, derived from an IL-7 transgenic mouse, which constitutively expresses MHC class II molecules whose expression can be slightly enhanced by IL-4. This pro-B-cell clone is able to process the native Hen Egg Lysozyme (HEL) protein into antigenic peptides capable of stimulating a class II–restricted T-cell hybridoma.

### RESULTS

Tumors obtained from our IL-7 transgenic mice were relatively monomorphic, and were mostly comprised of pro-B and pre-B cells (Fisher et al., 1993). Cells harvested from the tumors that arose in the 10-III-101 mouse were cloned *in vitro* using the B16.14 cell line as a stromal-cell feeder layer, as explained in Materials and Methods. The G12 clone is dependent upon cell contact with the B16.14 cells for its growth. This clone was analyzed first by cell-surface staining. As shown in Fig. 1(A), no expression of the myeloid markers Mac-1 and F4/80 was detected. On the contrary, G12 cells were found positive for B220, which is expressed throughout the B-cell differentiation pathway. Expression of CD25 was not detected. Figure 1(B) shows that G12 expresses BP-1, CD43, as well as Thy-1 antigens. Low levels of AA4.1 and λ5 molecules were also detected (in both cases, the peak median was shifted from 8.9 for the negative control to 14.6). The expression of AA4.1 is confined to early hemopoietic precursors (McKearn et al., 1985). The Thy-1 Ag has also been detected on pluripotent stem cells, as well as on bone-marrow-derived B-cell progenitors (Tidmarsh et al., 1989). The phenotype $B220^+$, $BP\text{-}1^+$, $HSA^+$, $CD43^+$, $\lambda5^+$, and $CD25^-$ is restricted to the pro-B/pre-BI cell stage, according to Rolink et al. (1994) and Hardy et al. (1994).

To define more accurately the differentiation stage of the G12 cells, we studied the rearrangement status of the Ig locus. This was analyzed by PCR using primers hybridizing 5′ to $D_H$ and 3′ to $J_H$ segments to detect $D_H$-$J_H$ rearrangements, and primers hybridizing 3′ to $D_H$ and 5′ to $J_H1$ segments to detect a germline fragment. As shown in Fig. 2, in G12 cells a 1.5-kb band corresponding to a germline D-$J_H$ fragment was detected, whereas no D-$J_H$ rearranged segments could be observed. The more mature 70Z/3 pre-B-cell line has both Ig alleles rearranged (Alt et al., 1984) and was used as positive control for D-$J_H$-rearranged fragments and negative control for germline DNA. The germline configuration of the Ig locus of G12 cells indicated that these cells are pro-B cells according to Rolink et al. (1994).

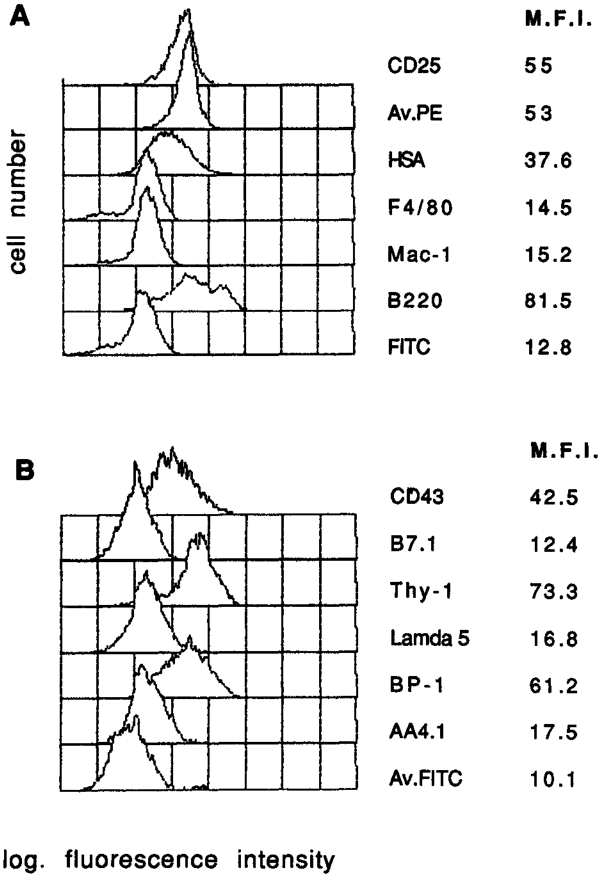

FIGURE 1. Cell-surface staining of G12 cells. (A) The staining of the rat mAb to HSA, F4/80, Mac-1, and B220 molecules was revealed with a FITC-conjugated anti-rat mAb. Analysis of CD25 expression was performed with a phycoerythrin-conjugated mAb. Means of fluorescence intensity are indicated. (B) The binding of the biotinylated mAbs to AA4.1, BP-1, Lamda-5, Thy-1, B7.1, and CD43 was visualized with avidin coupled to FITC.

We next assessed the expression of MHC class II molecules using the M5/114 mAb. This rat mAb detects $I\text{-}A^b$, $I\text{-}A^d$, $I\text{-}A^q$, and $I\text{-}E^k$ molecules but not $I\text{-}A^f$, $I\text{-}A^k$, and $I\text{-}A^S$ molecules (Bhattacharya et al., 1981). Because the G12 clone arose in a mouse issued from a crossing between an IL-7 transgenic mouse to DBA/2 (see Materials and Methods), at least one allele of its MHC is $H\text{-}2^d$. Indeed, G12 cells are stained with the M5/114 mAb, but the mean fluorescence intensity was approximately 10 times less than that obtained with the mature M12.4.1 B lymphoma cells (Fig. 3(A). The cell-surface detection

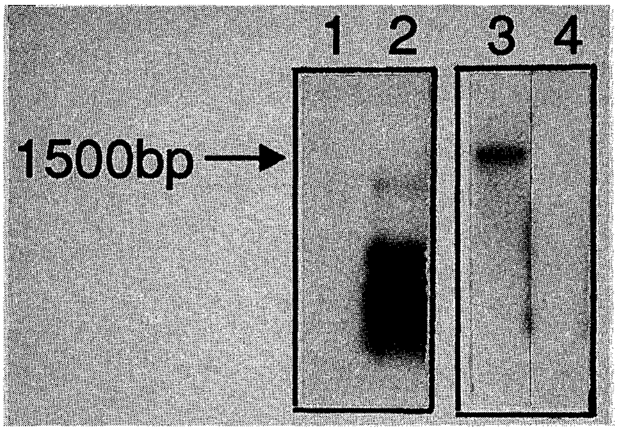

FIGURE 2. Southern blot analysis of PCR amplification. Amplified $D_H$-$J_H$-rearranged fragments (lanes 1 and 2) or germline fragments (lanes 3 and 4) were generated with DNA from G12 cells (lanes 1 and 3) or from 70Z/3 cells (lanes 2 and 4).

was much more sensitive when the rat M5/114 mAb was used instead of conventional mouse class II–specific mAbs, such as MKD6 or 40B (data not shown). We believe this may be due to a higher affinity of the xenogenic M5/114 mAb. Intracellular staining revealed a weak expression of the invariant chain (Ii) in G12 compared with M12.4.1 (Fig. 3 (B)). In these stainings, anti-CD8 H35.17 mAb served as an isotype control, all three mAbs being rat IgG2b. As already described (Polla et al., 1986), the chemically transformed 70Z/3 pre-B-cell line did not stain either at the cell-surface or intracellularly with M5/114 and anti-Ii mAb.

We next looked for MHC class II transcripts in G12 cells, using an I-$A_\beta$ probe. After S1 digestion of the RNA/DNA probe hybrids, a protected fragment yielding a band of approximately 440 bp was detected in the M12.4.1 positive control as well as with G12 RNA extract. No protected fragment could be detected with the negative control 70Z/3 nor with the B16.14 RNA extract. Indeed, the B16.14 cell line has been found negative for class II expression by flow cytometry, although this expression can be induced when the cells are grown at 33°C in the presence of IFN-γ (data not shown). Uncut probe (520 bp) remained visible in all four samples.

Having established that the pro-B G12 cells express MHC class II molecules, we next examined their ability to bind peptides either endogenously processed or exogenously provided. The presence of a cell-surface stable complex between MHC class II molecules and an antigenic peptide was assessed by the stimulation of IL-2 production by a relevant specific T-cell hybridoma. Figure 5(A) shows that the presentation of the HEL protein to the I-$E^d$-restricted HEL-specific G28 T-cell hybridoma is far less efficient by G12 than by M12.4.1 cells. Efficiency of presentation was similar only at the highest dose of HEL (10 mg/ml). For lower doses of antigen, T-cell response obtained with M12.4.1 cells remained at the plateau stage, whereas the T-cell response obtained with G12 cells decreased abruptly. Only doses ranging down to 1 mg/ml resulted in a significant T-cell response. When G12 cells were pretreated with 20% IL-4-containing supernatant for 48 hr prior to the T-cell stimulation test, a slight increase (7%) in the mean fluorescence intensity of class II molecules was detected by flow cytometry (data not shown). This pretreatment with IL-4 also increased the efficiency of antigen presentation (Fig. 5(A)). Similar results were obtained with the presentation of exogenously added synthetic (HEL (106–116) peptide (Fig. 5(B)). Curiously, the efficiency of peptide presentation by G12 cells never reached the high efficiency obtained by M12.4.1 cells, even at high peptide concentration (62.5 μg/ml).

### DISCUSSION

In this report, we have shown that MHC class II molecules can be expressed by an early B-cell precursor, namely, the G12 pro-B-cell clone. The level of class II expression by the G12 cells is about 10 times less than that found on a mature B-cell lymphoma line. Staining with the anti I-$A^d$ MKD6 mAB (not shown), as well as the efficient presentation of the I-$E^d$-restricted HEL(106–116) epitope indicates that both I-$A^d$ and I-$E^d$ molecules are expressed by G12 cells. The fact that the IL-7 transgene is under the control of the I-Eα promoter might suggest that class II-positive B cells would be autonomous of exogenous IL-7. However, G12 cells are totally dependent upon cell contact with B16.14 stromal cells and do not grow autonomously. The constitutive synthesis of IL-7 in our transgenic mouse results in the selective expansion of IL-7 responsive early B cells. Several other pro-B-cell clones have been derived from our IL-7 transgenic mice, and most do not express MHC class II molecules (Fisher et al., in press), indicating that the onset of MHC class II expression by G12 cells is not due to excessive signaling of IL-7 through the shared IL-7/4 receptor γ chain (Noguchi et al., 1993; Russel et al., 1993; Kondo et al., 1994). Recently, the expression of MHC class II molecules

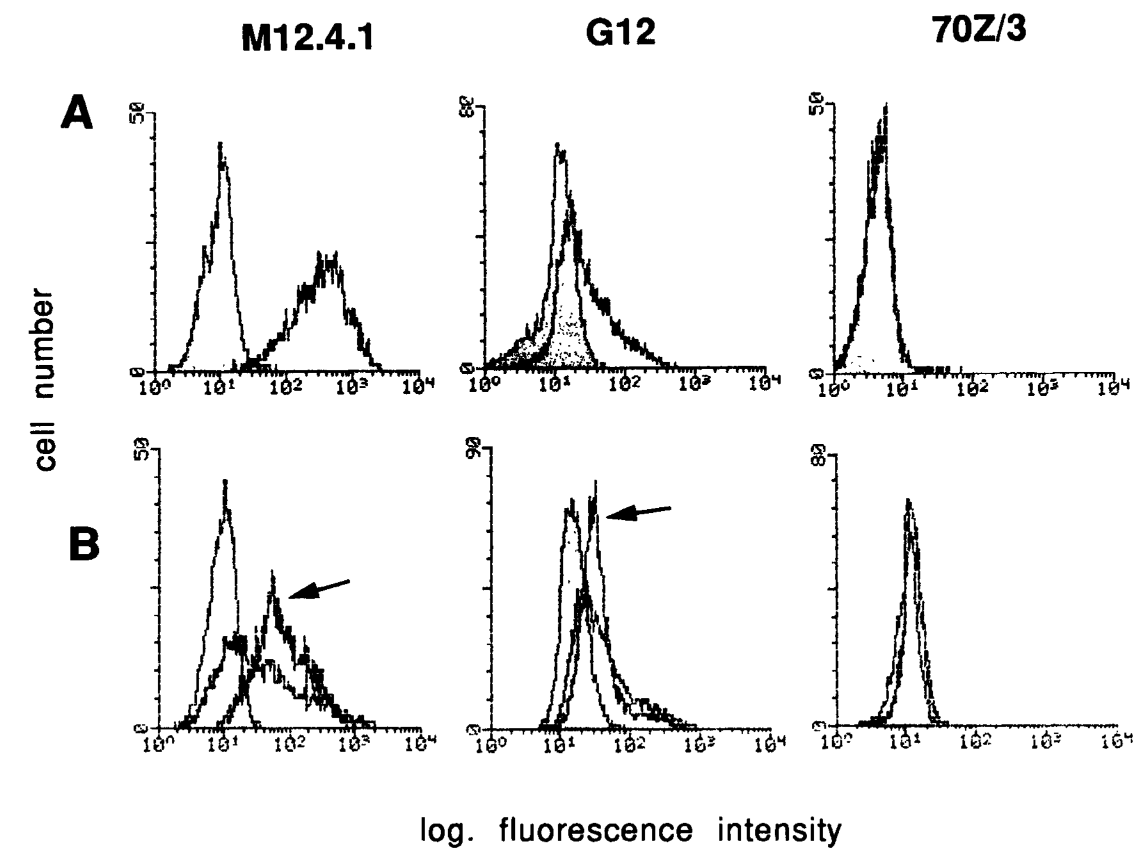

FIGURE 3. Flow cytometry analysis of MHC class II expression. (A) Cell-surface staining or (B) intracellular staining was performed with M12.4.1, G12, and 70Z/3 cells. MHC class II molecules were detected with M5/114 rat mAb, and invariant chain with In1 rat mAb. Profiles corresponding to In1 stain are indicated by an arrow.

by bone marrow B cells has been reinvestigated. Staining profiles obtained with fresh, polyclonal populations of pro-B cells, defined as B220$^+$ CD43$^+$, showed that class II expression was barely (Tarlinton, 1993) or not (Hayakawa et al., 1994) detectable. This may indicate that the onset of class II expression is an early and progressive event in the mouse B-cell maturation pathway; hence, only a small percentage of pro-B cells may be class II positive.

IL-4 is known for inducing MHC class II cell-surface expression in pre-B-cell lines as well as for increasing class II expression in resting peripheral B lymphocytes (Noelle et al., 1984; Polla et al., 1986). The presentation of the I-E$^d$-restricted HEL(106-116) epitope, which was increased when G12 cells were pretreated with IL-4, indicates that these pro-B cells can serve as APC. However, the presentation efficiency obtained with the G12 cells was always considerably less than that obtained with M12.4.1 cells. This was true with the native HEL protein as well as with added HEL(106–116) synthetic peptide, indicating that the difference observed is not due to

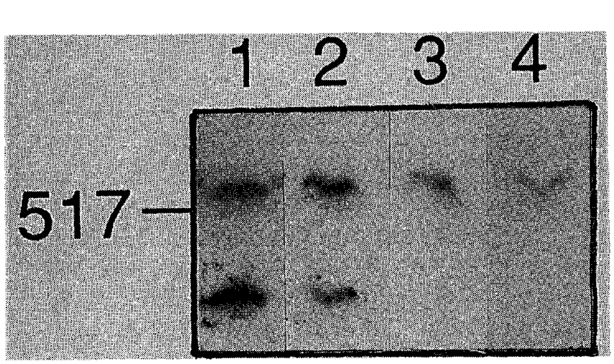

FIGURE 4. Detection of MHC class II transcripts by $S_1$ nuclease analysis. RNA extracts from M12.4.1, G12, 70Z/3, and B16.14 cells (lanes 1 to 4, respectively) were hybridized with a 520-bp $A_\beta$ probe. After S1 digestion, a 440-bp protected fragment was seen with M12.4.1 and G12 RNA extracts.

a major defect in the processing machinery of the G12 cells. One intriguing observation in our study is that, at high doses, the presentation of the native HEL protein by G12 cells is comparable to that obtained with the mature M12.4.1 B cells, whereas the optimal presentation by G12 cells of the exogenously provided HEL(106–116) peptide was always much inferior, even at high doses of synthetic peptide (see Figs. 4(A) and 4(B)). It has been shown that exogenously added peptides bind predominantly to preexisting MHC class II cell-surface molecules (Davidson et al., 1991). In mature B cells, class II molecules are continuously endocytosed and recycled to the cell surface (Harding et al., 1989; Reid and Watts, 1990; Salamero et al., 1990). During this recycling, MHC class II molecules may exchange peptides (Adorini et al., 1989). It may be that in G12 pro-B cells, class II molecules do not recycle properly, thereby rendering inefficient exchange of preexisting endogenous peptides for new exogenous peptides in MHC binding sites. In contrast, endocytosed processed antigens bind to newly synthesized MHC class II molecules in acidic intracellular compartments (Davidson et al., 1991). In mature B cells, a class II–specific peptide loading compartment has been described (Amigorena et al., 1994; Tulp et al., 1994; West et al., 1994). It remains to be seen if this specific compartment already exists in pro-B/pre-B cells.

In conclusion, although evidence obtained with class II–deficient mice suggests that MHC class II expression per se does not seem to play an important role in B-cell development (reviewed by Cardell et al., 1994), our data indicate that initial expression of the class II peptide-loading machinery may begin at the pro-B-cell stage. The relative inefficiency of exogenous Ag presentation by G12 pro-B cells suggests that further maturation of their peptide-loading compartment takes place during subsequent stages of B-cell differentiation. It has been shown that class II–restricted presentation of endogenously synthesized antigens is much more efficient than that of exogenously provided antigens (Calin-Laurens et al., 1992). Hence, class II–restricted presentation of exogenous and endogenous antigens, by pro-B cells onwards to mature B cells, may then occur in the bone marrow *in vivo*, where thymus-dependent as well as thymus-independent T cells have been found (Kubota et al., 1992; Hozumi et al., 1993), in normal as well as in pathological situations (Gravatt et al., 1993).

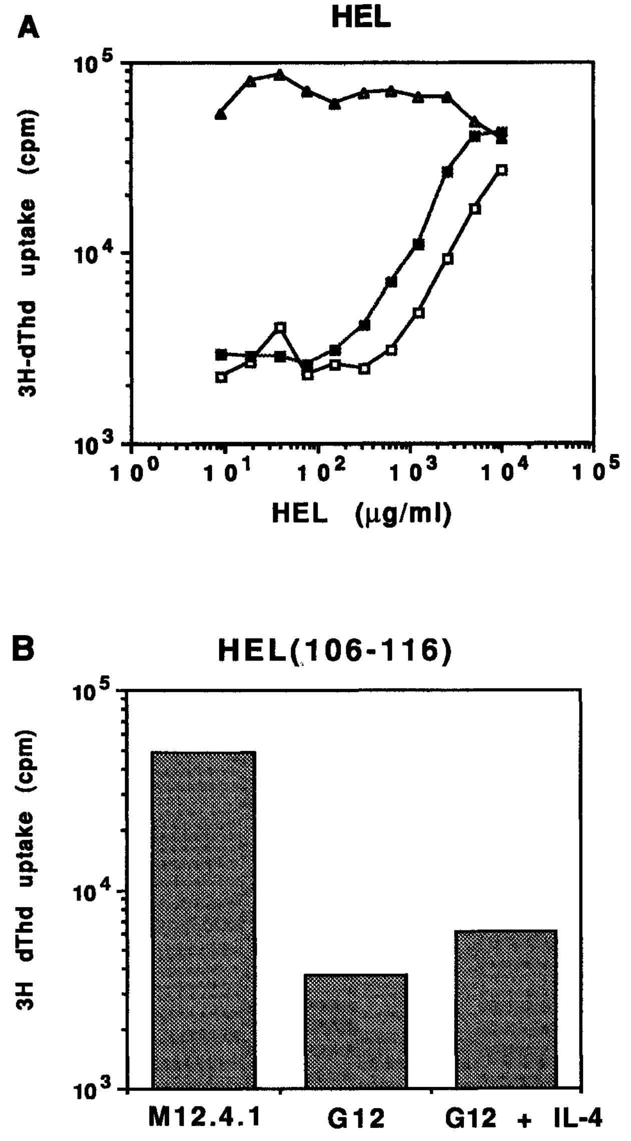

FIGURE 5. MHC class II–restricted presentation of the (A) native HEL or (B) HEL(106-116) synthetic peptide by G12 cells to the G28 T-cell hybridoma. M12.4.1 cells are represented by triangles, G12 cells by empty squares, and IL-4-treated G12 cells by filled squares. 3 x $10^4$ M12.4.1 cells or $10^5$ G12 cells were cocultured with $10^5$ G28 T cells for 24 hr in the presence of indicated concentrations of protein or 62.5 µg/ml of peptide. Supernatant was harvested and tested for the presence of IL-2 with CTLL.2 cells, as referred in Materials and Methods.

### MATERIALS AND METHODS

### Cell Lines

The 70Z/3 pre-B-cell line and the M12.4.1 mature B-lymphoma-cell line were originally generated from (C57BI/6 × DBA/2)$F_1$ ($BDF_1$) and Balb/c mice, respectively, and have been described elsewhere (Paige et al., 1978; Kim et al., 1979; Alt et al., 1984). In our transgenic line, the mouse IL-7 cDNA is under the control of the MHC class II Eα promoter (Fisher et al., 1993). The $BDF_1$ male founder mouse no. 10 was crossed with a normal C57BI/6 female. The transgenic progeny of this cross was again backcrossed onto C57BI/6 and then crossed onto DBA/2, giving rise to the 10-III-101 transgenic mouse in which a tumor arose from which the G12 clone had been derived. G12 cells have been cloned twice on IL-7-producing bone marrow stromal cells B16.14. This latter cell line has been established after immortalization of an adherent primary bone marrow stromal-cell culture from transgenic mice that had been infected with a recombinant retrovirus encoding a thermosensitive form of the SV40 T antigen (Fisher et al., 1993). The G28 HEL(106–116)-specific, I-$E^d$-restricted T-cell hybridoma has been described (Lombard-Platet et al., 1993).

### Immunofluorescence Analysis

To analyze the cell-surface expression of B220, Mac-1, and HSA, rat culture supernatants containing the monoclonal antibodies RA6B2, M1/70, and J11d were used, respectively. The rat antibodies were revealed with a 1:100 dilution of fluorescein isothiocyanate-conjugated goat anti-rat IgG (CALTAG Laboratories, San Francisco). The rat $IgG_{2b}$ M5/114.15.2 (ATCC TIB120) (Bhattacharya et al., 1981) monoclonal antibody was used for the detection of MHC class II molecules. Negative control was made by incubation of rat $IgG_{2b}$ H35.17 CD8-specific antibody (Pierres et al., 1982). Expression of CD25 was analyzed with a phycoerythrin-coupled mAb (Parmingen, San Diego). Negative control was performed with a streptavidin-phycoerythrin conjugate (Serotec, Oxford). The binding of the following biotinylated antibodies: AA4.1 (McKearn et al., 1985), BP-1 (Cooper et al., 1986), Thy-1 (Becton Dickinson, San Jose, CA), B7.1 (Pharmingen, San Diego) and LM34 (λ5-specific, a kind gift of Dr. A. Rolink (Karasuyama et al., 1994) were visualized with FITC-conjugated avidin (Becton Dickinson, San Jose). After staining, cells were fixed in 1% paraformaldehyde. Flow cytometric analyses were performed on a FACScan (Becton Dickinson, Mountain View, CA). Ethidium bromide (1 μg/ml) was added 2 min before analysis in order to stain dead cells in the samples. For intracellular staining, incubations were made in the presence of 0.3% saponin, and washes with 0.1% saponin. The rat In1.1 antibody was used to detect the presence of the invariant chain (Koch and Hammerling, 1982).

### PCR Assay

DNA was prepared as described (Haasner et al., 1994). The PCR conditions and oligonucleotide primers to amplify rearranged $D_H$-$J_H$ fragments, or germline fragments have been described (Gu et al., 1991), with slight modifications reported by Fisher et al. (1993). PCR samples were then separated on a 1% agarose gel and blotted onto a Hybond N membrane (Amersham Corp., Arlington Heights, IL). Filters were UV crosslinked, prehybridized, and then hybridized with a radioactive $J_H4$ oligonucleotide probe (5′-AGGAACCTCAGTCACCGGATC CGT-3′) (Haasner et al., 1994).

### Cell Culture and T-Cell Stimulation Assay

Cells were grown in DMEM (Gibco BRL, Grand Island, NY) supplemented as previously described (Lombard-Platet et al., 1993). B16.14 were maintained at 33°C. Before using them as feeder layer cells, they were treated for 45 min at 37°C with 25 μg/ml mitomycin C (Sigma Chemicals, St. Louis), and then extensively washed and seeded at 37°C at the ratio of approximately five B16.14 for one G12 cell. IL-4 containing supernatant was harvested from X63 myeloma cells transfected with an expression vector encoding mouse IL-4 cDNA (Karasuyama and Melchers, 1988). Grade I HEL was purchased from Sigma Chemicals. HEL (106–116) synthetic peptide was synthesized in this Institute. T-cell stimulation assays were performed as previously described (Lombard-Platet et al., 1993). The Interleukin-2 content of culture supernatants was measured using the CTLL-2 line. Proliferation of CTLL-2 cells was evaluated by incorporation of tritiated thymidine (DuPont NEN, Boston).

### Detection of MHC Class II mRNA

RNA were prepared with the method of Chomczynski and Sacchi (1987). A 5′-end-labeled probe was synthesized by primer extension. 10 pmoles of primer-oligonucleotide (5′-GGAAGATGTTGTCCA CAAAGCAGAT-3′) was labeled and annealed to a single-stranded $A^k_\beta$ template (a kind gift from Drs.

D. Mathis and C. Benoist). The template/probe hybrid was digested with 20 U Pst1. S1 mapping was performed as described (Viville et al., 1991).

### ACKNOWLEDGMENTS

The authors are grateful to Dr. A. Rolink for the gift of LM34 mAb and Dr. V. Kouskoff for kindly providing the single-stranded $A^k_\beta$ template. This work was supported in part by grants from l'Association pour la Recherche sur le Cancer (ARC no. 6064).

*(Received August 24, 1994)*

*(Accepted November 18, 1994)*

### REFERENCES

Adorini L., Appella E., Doria G., Cardinaux F., and Nagy Z.A. (1989). Competition for antigen presentation in living cells involves exchange of peptides bound by class II MHC molecules. Nature **342**: 800–802.

Alt F.W., Yancopoulos G.D., Blackwell, T.K., Wood, C., Thomas, E., Boss M., Coffman R., Rosenberg N., Tonegawa S., and Baltimore D. (1984). Ordered rearrangement of immunoglobulin heavy chain variable region segments. (EMBO J. **3**: 1209–1219.

Amigorema S., Drake J.R., Webster P., and Mellamn I. (1994). Transient accumulation of new class II MHC molecules in a novel endocytic compartment in B lymphocytes. Nature **369**: 113–120.

Bhattacharya A., Dorf M.E., and Springer T.A. (1981). A shared alloantigenic determinant on la antigens encoded by the I-A and I-E sub-regions: evidence for I region gene duplication. J. Immunol. **127**: 2488–2495.

Calin-Laurens V., Forquet F., Lombard-Platet S., Bertolino P., Chrétien I., Trescol-Biémont M.-C., Gerlier D., and Rabourdin-Combe C. (1992). High efficiency of endogenous antigen presentation by MHC class II molecules. Int. Immunnol. **10**: 1113–1121.

Cardell S., Merkenschlager M., Bodmer H., Chan S., Cosgrove D., Benoist C., and Mathis D. (1994). The immune system of mice lacking conventional MHC class II molecules. Adv. Immunol. **55**: 423–440.

Chomczynski P., and Sacchi N. (1987). Single step method of RNA isolation by acid guanidinium thiocyanate phenol chloroform extraction. Anal. Biochem. **162**: 156–159.

Cooper M.D., Mulvaney D., Coutinho A., and Cazenave P.-A. (1986). A novel cell-surface molecule on early B-lineage cells. Nature **321**: 616–618.

Dasch J.R., and Jones P. (1986) Independent regulation of IgM, IgD, and Ia antigen expression in cultured immature B lymphocytes. J. Exp. Med. **163**: 938–951.

Davidson H.W., Reid P.A., Lanzaveccia A., and Watts C. (1991). Processed antigen binds to newly synthesized MHC class II molecules in antigen-specific B lymphocytes. Cell **67**: 105–116.

Fisher A.G., Burdet C., Bunce C., Merkenschlager M., and Ceredig R. (1995). Lymphoproliferative disorders in IL-7 transgenic mice: Expansion of immature B cells which retain macrophage potential. Int. Immunol. (in press).

Fisher A., Burdet C., LeMeur M., Haasner D., Gerber P., and Ceredig R. (1993). Lymphoproliferative disorders in an IL-7 transgenic mouse line. Leukemia (Sup. 2): S66–S68.

Gravatt L.C., Chaffee S., Hebert M.E., Halperin E.C., Friedman H.S., and Kurtzberg J. (1993). Efficacy and toxicity of 9-beta-D-arabinofuranosylguanine (araG) as an agent to purge malignant T cells from murine bone marrow: application to an in vivo T-leukemia model. Leukemia **7**: 1261–1267.

Gu H., Kitamura D., and Rajewsky K. (1991). B cell development regulated by gene rearrangement: Arrest of maturation by membrane-bound Dμ protein and selection of $D_H$ element reading frames. Cell **65**: 47–54.

Haasner D., Rolink A., and Melchers F. (1994). Influence of surrogate L chain on $D_H$-$J_H$ reading frame 2 suppression in mouse precursor B cells. Int. Immunol. **6**: 21–30.

Harding C.V., Roof R.W., and Unanue E.R. (1989). Turnover of Ia-peptide complexes is facilitated in viable antigen-presenting cells: Biosynthesis turnover of Ia vs. peptide exchange. Proc. Natl. Acad. Sci. USA **86**: 4230–4234.

Hardy R.R., Carmack C.E., Li Y.S., and Hayakawa K. (1994) Distinctive developmental origins and specificities of murine CD5 + B cells. Immunol. Rev. **137**: 91–118.

Hayakawa K., Tarlinton D., and Hardy R.R. (1994). Absence of MHC class II expression distinguishes fetal from adult B lymphoiesis in mice. J. Immunol. **152**: 4801–4807.

Hozumi K., Masuko T., Nishimura T., Habu S., and Hashimoto Y. (1993). Characterisation of the T cells in aged rat bone marrow. Immunol. Lett. **36**: 137–143.

Karasuyama H., and Melchers F. (1988). Establishment of mouse cell lines which constitutively secrete large quantities of interleukin 2, 3, 4, or 5. Eur. J. Immunol. **18**: 97–104.

Karasuyama H., Rolink A., Shinkai Y., Young F., Alt F.W., and Melchers F. (1994). The expression of VpreB/λ5 surrogate light chain in early bone marrow precusor B cells of normal and B cell-deficient mutant mice. Cell **77**: 133–143.

Kim K.J., Kanellopoulos-Langevin, Merwin R. M., Sachs D.H., and Asofsky R. (1979). Establishment and characterisation of Balb/c lymphoma cell lines with B cell properties. J. Immunol. **122**: 549.

Koch N., and Hammerling G.J. (1982). Ia invariant chain detected on lymphocyte surfaces by monoclonal antibody. Nature **299**: 644–645.

Kondo M., Takeshita T., Higuchi M., Nakamura M., Sudo T., Nishikawa S.-I., Sugamura K. (1994) Functional participation of the IL-2 receptor γ chain in IL-7 receptor complexes. Science **263**: 1453–1454.

Kubota H., Okazaki H., Onuma M., Kano S., Hattori M., and Minato N. (1992). $CD3^+4^-8^-$ alpha beta T cell population with biased T cell receptor V gene usage. Presence in bone marrow and possible involvement of IL-3 for their extrathymic development. J. Immunol. **149**: 1143–1150.

Lombard-Platet S., Bertolino P., Deng H., Gerlier D., and Rabourdin-Combe C. (1993). Inhibition by chloroquine of the class II major histocompatibility complex-restricted presentation of endogenous antigens varies according to the cellular origin of the antigen-presenting cells, the nature of the T-cell epitope, and the responding T cell. Immunology **80**: 566–573.

McKearn J.P., McCubrey J., and Fagg B. (1985). Enrichment of hematopoietic precursor cells and cloning of multipotential B-lymphocyte precursors. Proc. Natl. Acad. Sci. USA **82**: 7414–7418.

Miki N., Hatano M., Wakita K., Imoto S., Nishikawa S.-I., and Tokuhisa T. (1992). Role of I-A molecules in early stages of B cell maturation. J. Immunol. **149**: 801–807.

Noelle R., Krammer P.H., Ohara J., Uhr J.W., and Vitetta E.S. (1984). Increased expression of Ia antigens on resting B cells: An additional role for B-cell growth factor. Proc. Natl. Acad. Sci. USA **81**: 6149–6153.

Noguchi M., Nakamura Y., Russel S.M., Ziegler S.F., Tsang M., Cao X., and Leonard W.J. (1993). Interleukin-2 receptor γ

chain: A functional component of the interleukin-7 receptor. Science **262**: 1877–1880.

Paige C.J., Kincade P.W., and Ralph P. (1978). Murine B cell leukemia line with inducible surface immunoglobulin expression. J. Immunol. **121**: 641–647.

Pierres M., Goridis C., and Golstein P. (1982). Inhibition of murine T-cell mediated cytolysis and T cell proliferation by a rat monoclonal antibody immunoprecipitating two lymphoid cell surface polypeptides of 94,000 and 180,000 molecular weight. Eur. J. Immunol. **12**: 60–69.

Polla B.S., Poljak A., Ohara J., Paul W.E., and Glimcher L.H. (1986). Regulation of class II gene expression: Analysis in B cell stimulatory factor 1-inducible murine pre-B cell lines. J. Immunol. **137**: 3332–3337.

Reid P.A., and Watts C. (1990). Cycling of cell-surface MHC glycoproteins through primaquine-sensitive intracellular compartments. Nature **346**: 655–657.

Rolink A., Kudo A., Karasuyama H., Kikuchi Y., and Melchers F. (1991). Long-term proliferating early pre B cell lines and clones with the potential to develop to surface Ig-positive, mitogen reactive B cells in vitro and in vivo. EMBO J **10**: 327–336.

Rolink A., Karasuyama H., Haasner D., Grawunder U., Martensson I.-L., Kudo A., and Melchers F. (1994). Two pathways of B-lymphocyte development in mouse bone marrow and the roles of surrogate L chain in this development. Immunol. Rev. **137**: 185–202.

Russel S.M., Keegan A.D., Harada N., Nakamura Y., Noguchi M., Leland P., Friedman M.C., Miyajima A., Puri R.K., Paul W. E., Leonard W.J. (1993). Interleukin-7 receptor $\gamma$ chain: A functional component of the interleukin-4 receptor. Science **262**: 1880–1883.

Salamero J., Humbert M., Cosson P., and Davoust J. (1990). Mouse B lymphocyte specific endocytosis and recycling of MHC class II molecules. EMBO J. **9**: 3489–3496.

Tarlinton D. (1993). Direct demonstration of MHC class II surface expression on murine pre-B cells. Int. Immunol. **5**: 1629–1635.

Tidmarsh, Heimfeld S., Whitlock C.A., Weissman I., and Müller-Sieburg C.E. (1989). Identification of a novel bone marrow–derived B-cell progenitor population that coexpresses B220 and Thy-1 and is highly enriched for Abelson leukemia virus targets. Mol. Cell. Biol. **9**: 2665–2671.

Tulp A., Verwoerd D., Dobberstein B., Ploegh H.L., and Pieters J. (1994). Isolation and characterisation of the intracellular MHC class II compartment. Nature **369**: 120–126.

Viville S., Jongeneel V., Koch W., Mantovani R., Benoist C., and Mathis D. (1991). The E$\alpha$ promoter: A linker-scanning analysis. J. Immunol. **146**: 3211–3217.

West M. A., Lucocq J. M., and Watts C. (1994). Antigen processing and class II MHC peptide-loading compartments in human B-lymphoblastoid cells. Nature **369**: 147–150.

